# Dog and Cat Ownership Predicts Adolescents’ Mental Well-Being: A Population-Based Longitudinal Study

**DOI:** 10.3390/ijerph17030884

**Published:** 2020-01-31

**Authors:** Kaori Endo, Syudo Yamasaki, Shuntaro Ando, Takefumi Kikusui, Kazutaka Mogi, Miho Nagasawa, Itsuka Kamimura, Junko Ishihara, Miharu Nakanishi, Satoshi Usami, Mariko Hiraiwa-Hasegawa, Kiyoto Kasai, Atsushi Nishida

**Affiliations:** 1 Department of Psychiatry and Behavioral Sciences, Tokyo Metropolitan Institute of Medical Science, 2-1-6 Kamikitazawa, Setagaya-ku, Tokyo 156-8506, Japan; endo-kr@igakuken.or.jp (K.E.); nakanishi-mh@igakuken.or.jp (M.N.); nishida-at@igakuken.or.jp (A.N.); 2 Department of Neuropsychiatry, Graduate School of Medicine, University of Tokyo, 7-3-1 Hongo, Bunkyo-ku, Tokyo 113-8655, Japan; sandou-tky@umin.ac.jp (S.A.); kasaik-tky@umin.net (K.K.); 3 Department of Animal Science and Biotechnology, Azabu University, 1-17-71 Fuchinobe, Chuo-ku, Sagamihara-shi, Kanagawa 252-5201, Japan; takkiku@carazabu.com (T.K.); mogi@carazabu.com (K.M.); nagasawa@carazabu.com (M.N.); i.kamimura.09@carazabu.com (I.K.); 4 Department of Food and Life Science, Azabu University, 1-17-71 Fuchinobe, Chuo-ku, Sagamihara-shi, Kanagawa 252-5201, Japan; j-ishihara@azabu-u.ac.jp; 5 Graduate School of Education, The University of Tokyo, 7-3-1 Hongo, Bunkyo-ku, Tokyo 113-0033, Japan; usami@ct.u-tokyo.ac.jp; 6 School of Advanced Science, SOKENDAI (Graduate University for Advanced Studies), Shonan Village, Hayama, Kanagawa 240-0193, Japan; hasegawa_mariko@soken.ac.jp

**Keywords:** pets, dogs, cats, cohort studies, adolescent, well-being

## Abstract

A potential association between pet ownership and mental well-being is suggested, but there is a shortage of high-quality longitudinal studies that consider probable differences among different species. We aimed to examine whether ownership of the most popular pets (dogs and cats) would predict mental well-being. The Tokyo Teen Cohort (TTC), a prospective population-based birth cohort study, had dog and cat ownership data at age 10 and mental well-being score at ages 10 and 12 from 2584 adolescents. Linear regression analysis with adjusting for covariates showed that dog ownership had a positive effect on mental well-being compared to no dog ownership, however, cat ownership had a negative effect compared to no cat ownership. Two-factor mixed-design analysis of variance showed that dog ownership predicted maintained mental well-being, while cat ownership predicted progressing decline of mental well-being. Thus, dog and cat ownership may have different effects on adolescents’ mental well-being, implying that the underlying mechanisms that are activated by these types of ownership may differ.

## 1. Introduction

Adolescence is the phase of life between late childhood and adulthood [1]. It is a unique developmental stage during which an individual is constantly being shaped and influenced by their environment [2]. The effects of the environment are sometimes irreversible on mental and emotional development as well as physical maturation [3,4]. Positive youth development can lead to a healthy and successful adulthood [5]. Providing an environment that supports positive youth development is thus beneficial for not only adolescents, but also for individuals of all ages [6].

Pet ownership may be an important environmental factor for mental well-being among adolescents as several studies have suggested an association between pet ownership and mental well-being among adolescents [7,8,9]. However, this association is not limited to adolescence. Companionship with pets may be important for positive mental health and well-being [10]. Connection with pets provides benefits to those with mental health problems by offering emotional support [11]. Moreover, pet ownership is a modifiable environmental factor [12,13] because we can choose whether we own pets or not. However, the results of previous studies have been controversial, and a recent systematic review showed there is a shortage of high-quality and longitudinal studies that consider probable differences among different species [14]. Although dogs and cats are among the most popular companion animals in the world, they may have different effects on the mental well-being of humans, which are activated through different underlying mechanisms depending on the type of ownership. A recent study showed that the human–dog interaction through dogs’ human-like gazing behavior increased human oxytocin [15], which has received increasing attention for its role in promoting positive social behavior and stress regulation, and its potential as a therapeutic intervention for addressing various aspects of psychiatric disorders [16,17]. Conversely, a recent meta-analysis revealed that the presence of the parasite *Toxoplasma gondii* in the human body, which may be transmitted from cats to humans, is significantly associated with increased risk of traffic accidents and suicide attempts among those infected [18]. *T. gondii* alters host’s behavior [19], and the oocytes seem to be a risk factor for developing schizophrenia [20]. Thus, cat ownership in childhood may be related to later schizophrenia risk [21]. On the other hand, the ALSPAC cohort study in the UK showed that cat ownership in pregnancy and childhood did not increase the risk of adolescent psychotic experiences [22]. Given these results, it is possible that the impact of dog and cat ownership on adolescents’ mental well-being may be more complex than it seems.

This study aimed to examine the effect of dog and cat ownership on the longitudinal trajectory of the mental well-being of adolescents using data from a population-based birth cohort study (The Tokyo Teen Cohort), while taking into account a wide range of confounding variables and considering the differences among the two analyzed species. To the best of our knowledge, this is the first study that longitudinally analyzes the effect of pet ownership, taking into account species difference while also analyzing data from a prospective and population-based birth cohort study with a large sample.

## 2. Materials and Methods 

### 2.1. Data and Samples

This study was part of the Tokyo TEEN Cohort (TTC) project (for the protocol, see [23]), an ongoing, prospective, and population-based birth cohort study on adolescents and their primary caregivers. Briefly, the TTC aimed to investigate the health and development of adolescents, and its details are described elsewhere [24,25,26]. In the first time point of the study, a sample of 3171 households with adolescents aged 10 years (i.e., born between September 2002 and August 2004) was obtained from 3 municipalities (Chofu, Mitaka, and Setagaya) in Tokyo, Japan, by random sampling from the basic resident register. When children were aged 12 years, 3007 households participated in the second time point of the study (follow-up rate: 94.8%). Trained interviewers obtained written informed consent from the adolescents’ primary caregivers, asked adolescents and their caregivers to complete a set of questionnaires, conducted a semi-structured interview, and measured anthropometric data (height, weight, and grip). The study protocol of the TTC was approved by the institutional review boards from the Tokyo Metropolitan Institute of Medical Science (Approval number: 12–35), SOKENDAI (Graduate University for Advanced Studies (2012002)) and the University of Tokyo (10057).

### 2.2. Variables

Adolescents were interviewed to determine whether they have any pets in their home, “Do you have any pets?” at age 10. Their responses were coded in 2 dichotomized variables as 1) own (1) or not own (0) for a dog or 2) own (1) or not own (0) for a cat.

### 2.3. Outcome

Mental well-being was assessed at ages 10 and 12, using the self-report questionnaire which was a 5-item World Health Organization Well-Being Index (WHO5) [27,28]. Each item assessed the degree of well-being over the past 2 weeks on a 6-point Likert-type scale ranging from 0 (at no time) to 5 (all of the time). Total scores derived from the WHO5 ranged from 0 to 25, with higher scores indicating better psychological well-being. The total raw score, ranging from 0 to 25, was multiplied by 4 to obtain the final score, with 0 representing the worst imaginable well-being and 100 representing the best imaginable well-being. The WHO5 scale was used in the original format without modifications. All existing language versions of this questionnaire are available on the website [29].

### 2.4. Covariates 

The covariates included were sex, age, parental age, parental educational level, annual household income, and the number of siblings. This information was collected at age 10. We adjusted for multiple confounders which were applied in the previous studies, including sex [30,31,32], age [31], parental age [22,32], parental educational level [22,30,32], annual household income (in relevance to social class and work status) [22,30,31,32], and number of siblings (in relevance to presence of older siblings, number of people in the household, and household crowding) [22,31,32]. 

### 2.5. Statistical Analysis

Linear regression analysis was performed to estimate the associations between pet ownership at age 10 and well-being at age 12. We calculated the non-standardized *B*s (multiple regression coefficients) of dog-ownership and cat-ownership. We then adjusted for the covariates. Two-factor mixed-design analysis of variance (ANOVA) for pet-owner types (non-dog/cat owners, dog owners (owned no cats), cat owners (owned no dogs)) and 2 time points (ages 10 and 12) was performed. The group who owned both dogs and cats was excluded because their number was too low, and the sample was not representative (*n* = 9). The significance level (*α*) was set to 0.05 for a 2-sided test. All statistical analyses were conducted using IBM SPSS Statistics for Windows, version 24.0.0.1 (IBM Corp., Armonk, NY, USA).

## 3. Results

Of the 3171 initially enrolled households, 2584 (81.5%) were included in our final analytic sample. Participants who had missing data on pet ownership, well-being at age 10 and 12, sex, age (months), parental age, parental educational level, annual household income, and the number of siblings were excluded from the present analyses. There were no differences among the excluded and included subjects in terms of dog ownership (*χ*^2^ = 0.20, *p* = 0.650), well-being at age 10 (*χ*^2^ = 37.36, *p* = 0.167), well-being at age 12 (*χ*^2^ = 19.67, *p* = 0.943), sex (*χ*^2^ = 0.33, *p* = 0.566), age in months (*χ*^2^ = 15.46, *p* = 0.563), mother’s age (*χ*^2^ = 38.34, *p* = 0.115), father’s age (*χ*^2^ = 31.07, *p* = 0.843), mother’s educational level (*χ*^2^ = 10.15, *p* = 0.071), and father’s educational level (*χ*^2^ = 5.54, *p* = 0.354), although there were differences in terms of cat ownership (*χ*^2^ = 5.55, *p* = 0.018), annual household income (*χ*^2^ = 228.80, *p* < 0.001), and number of siblings (*χ*^2^ = 16.45, *p* = 0.012). Cat ownership was higher in excluded subjects, and annual household income and number of siblings were higher in included subjects.

Demographic characteristics of participants are shown in Table 1. Approximately 10% of adolescents owned dogs, and 4% owned cats. The results of linear regression analysis showed that dog ownership at age 10 predicted better well-being at age 12 compared to no dog ownership (*B* = 2.61, 95% CI: 0.17–5.05, *p* = 0.036), while cat ownership at age 10 predicted worse well-being at age 12 compared to no cat ownership (*B* = −5,65. 95% CI: −9.26–−2.03, *p* = 0.002). The effect remained significant after adjusting for covariates (dog: *B* = 2.45, 95% CI: 0.19–4.71, *p* = 0.033; cat: *B* = 6.14, 95% CI: −9.49–−2.79, *p* < 0.001). These results are shown in Table 2. Among boys, dog ownership at age 10 predicted better well-being at age 12 compared to no dog ownership (*B* = 3.32, 95%CI: 1.00–7.86, *p* = 0.011), while cat ownership at age 10 predicted worse well−being at age 12 compared to no cat ownership (*B* = −6.55, 95%CI: −11.60–−2.45, *p* = 0.010). The effect remained significant after adjusting for covariates (dog: *B* = 4.04, 95%CI: 0.90–7.18, *p* = 0.012,; cat: *B* = −7.03, 95%CI: −11.60–−2.45, *p* = 0.003). Among girls, neither dog ownership nor cat ownership at age 10 predicted better nor worse well-being at age 10 (dog: *B* = 0.64, 95%CI: −2.84–4.12, *p* = 0.719; cat: *B* = −4.63, 95%CI: −9.89–0.62, *p* = 0.084). Adjusting for covariates did not change the results for dog ownership (*B* = 0.65, 95%CI: −2.61–3.91, *p* = 0.696) but did for cat ownership (*B* = −5.34, 95%CI: −10.27–−0.41, *p* = 0.034).

**Figure 1:**
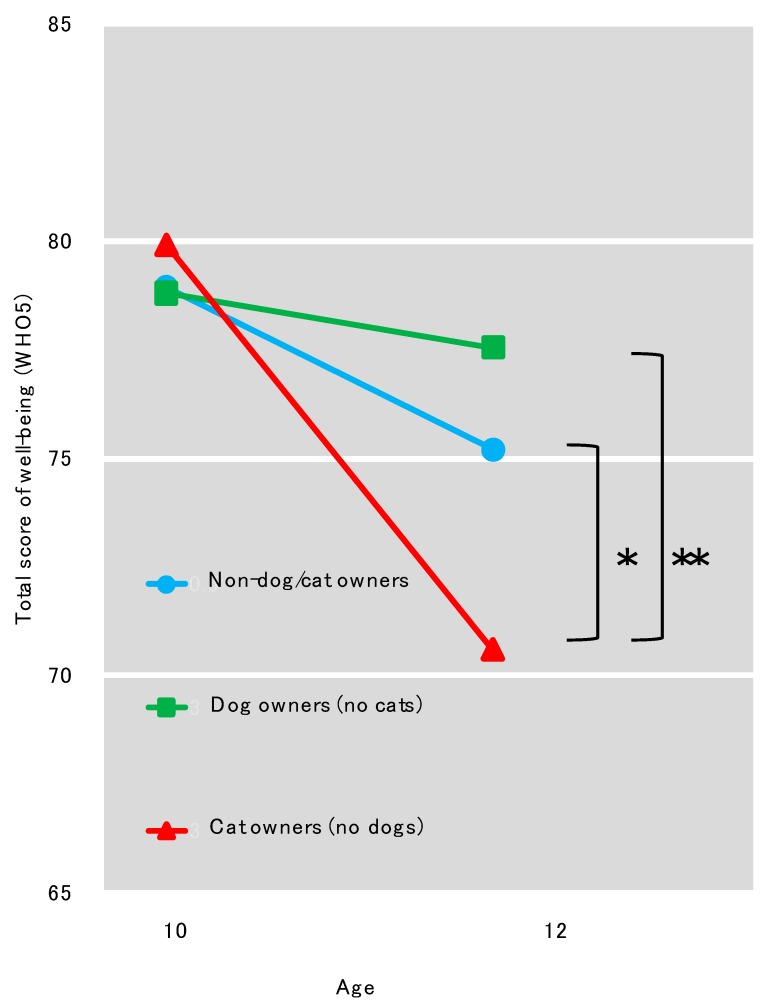
Averages of well-being (WHO5) at ages 10 and 12 among non-dog/cat owners, dog owners, and cat owners (* *p* < 0.05, ** *p* < 0.01). Two-way mixed-design analysis of variance (ANOVA) showed significant interaction of time points and owner types (*F* (2, 2572) = 6.78, *p* = 0.001). Simple main effect of owner types was not significant at age 10 (*F* (2, 2572) = 0.18, *p* = 0.835), but it was significant at age 12 (*F* (2, 2572) = 6.61, *p* = 0.001). Bonferroni adjustments were administered for multiple comparisons and found significant pairs at age 12 as follows: cat owners (owned no dogs) and non-dog/cat owners (*p* = 0.017) and cat owners (owned no dogs) and dog owners (owned no cats) (*p* = 0.001). Other pairs were not significant. The simple main effect of time points was significant in non-dog/cat owners (*F* = 85.55, *p* < 0.001) and cat owners (owned no dogs) (*F* = 26.21, *p* < 0.001) but not significant in dog owners (owned no cats) (*F* = 1.38, *p* = 0.240).

Two-way mixed-design ANOVA showed significant interaction of time points and owner types (*F* (2, 2572) = 6.78, *p* = 0.001). Simple main effect of owner types was not significant at age 10 (*F* (2, 2572) = 0.18, *p* = 0.835), but it was significant at age 12 (*F* (2, 2572) = 6.61, *p* = 0.001). Bonferroni adjustments were administered for multiple comparisons and found significant pairs at age 12 as follows: cat owners (owned no dogs) and non-dog/cat owners (*p* = 0.017) and cat owners (owned no dogs) and dog owners (owned no cats) (*p* = 0.001). Other pairs were not significant. The simple main effect of time points was significant in non-dog/cat owners (*F* = 85.55, *p* < 0.001) and cat owners (owned no dogs) (*F* = 26.21, *p* < 0.001) but not significant in dog owners (owned no cats) (*F* = 1.38, *p* = 0.240). The related result below is shown in Figure 1. Among boys, a significant interaction of time points and owner types was also found (*F* (2, 1357) = 6.202, *p* = 0.002). Simple main effect of owner types was not significant at age 10 (*F* (2, 1357) = 0.189, *p* = 0.828), but it was significant at age 12 (*F* (2, 1357) = 6.284, *p* = 0.002). Bonferroni adjustments were administered for multiple comparisons and found significant pairs at age 12 as follows: dog owners (owned no cats) and non-dog/cat owners (*p* = 0.020), and dog owners (owned no cats) and cat owners (owned no dogs) (*p* = 0.002). Other pairs were not significant. The simple main effect of time points was significant in no dog or cat owners (*F* = 33.08, *p* < 0.001) and cat owners (owned no dogs) (*F* = 14.21, *p* < 0.011) but was not significant in dog owners (owned no cats) (*F* = 0.30, *p* = 0.581). Among girls, the interaction of time points and owner types was not significant (*F* (2, 1212) = 1.810, *p* = 0.164). Simple main effects of owner types was neither significant at age 10 (*F* (2, 1212) = 0.029, *p* = 0.972) nor at age 12 (*F* (2, 1212) = 1,704, *p* = 0.182). The simple main effect of time points was significant in all groups (non-dog/cat owners: *F* = 54.63, *p* < 0.001; cat owners: *F* = 12.04, *p* = 0.001; dog owners: *F* = 5.28, *p* = 0.022).

## 4. Discussion

This is the first study to investigate the different effects of dog ownership and cat ownership on adolescents’ well-being, adjusting for various demographic and socioeconomic variables using a large-sample, longitudinal, population-based study. The prevalence of dog/cat ownership in this study was consistent with a previous large-scale Japanese study [33]. Dog ownership at age 10 was associated with increased well-being at age 12 compared to no dog ownership, and cat ownership at age 10 was associated with decreased well-being at age 12 compared to no dog ownership. These results were also the same after adjusting for covariates, including socio-demographic factors. 

Previous studies have shown that the mental well-being of an individual in their adolescence has a long-lasting impact on the individual’s later life [2,34], even though mental well-being was shown to generally decline throughout adolescence [30]. Previous studies have revealed that the life-long trajectory of well-being is U-shaped; it declines through the teen years to young adulthood, hits the bottom around the 40s or 50s, and increases thereafter [30,35]. In our study, we confirmed that well-being declined from age 10 to age 12 and identified the preventive effect of dog ownership on the decline of well-being. On the other hand, we also identified that the well-being of the cat owners’ group significantly declined compared with the 2 other groups (dog owner group and non-dog/cat owner group). 

Our results suggested that the effect of dog and cat ownership on adolescent well-being may have different underlying mechanisms. One factor may be the owner’s physical activity with their pet. Dog owners often go walking with their pet [36]. Dog walking brought adolescents 7–8% more physical active minutes per day [37] and has a benefit for children’s overweight or obesity [38]. However, cat owners may not go for a walk with their pets but may play with pets indoors. This could cause the difference in time length and intensity of owner’s physical activity and lead to higher/lower well-being. As previous research shows, some parents own a pet because they want to teach their children responsibility and kindness [39]. Children from household with pets may learn responsibilities that benefit the development of their well-being. This point should be also considered in future studies. Deepening this discussion with a biological view, we know that oxytocin is a neuropeptide, receptors for which are distributed in one’s brain. Oxytocin relates to trust in other humans [40] and modulates our sociality [41]. Further, through social interaction, oxytocin suppresses cortisol concentration, which is a response to stress [42]. Dog gaze has been shown to increase the oxytocin level in owner’s urine [15], which means the dog may increase oxytocin in its owner, promote social bonding, and decrease stress levels. However, a similar testing method was not used for cats, therefore, we should be careful how we interpret the results of cat ownership in this study. Future studies should aim to investigate the effects of cats on humans based on useful methods in previous studies [43,44]. With respect to cats, a previous study about the risk of childhood cat ownership on schizophrenia in later life, mentioned the possibility of *T. gondii* infection from cats [21]. Moreover, we know that *T. gondii* is a parasite which affects warm-blooded animals [18]. Their primary hosts are cats [22]. *T. gondii* affects the brain through inflammation or changes in the microbiome [45]. As several experiments have shown, *T. gondii* alters the behavior of rodents, making them easier prey for cats [19]. Mice infected by *T. gondii* once lose innate aversion for cat’s urine permanently even after the infection has been removed [46]. In humans, *T. gondii* may be associated with an elevated risk for mental health issues, such as psychosis-like symptoms, bipolar disorder, violence, suicide attempts, anxiety disorder, and obsessive disorder [45]. In summary, a cat can be an infection source of *T. gondii* in its owner, causing brain dysfunction by inflammation or alteration in the microbiome, and leading to psychiatric symptoms. A future TTC study will attempt to answer these remaining questions of psychological, physiological, and biological mechanisms.

Our results showed that not all types of pet ownership enhance adolescents’ well-being, and that there can be substantial differences based on the species owned. Compared with cat ownership, dog ownership seemed to be more beneficial for maintaining well-being across adolescence. A previous experimental study reported that interaction with a therapy dog during 20 minutes improved well-being among students [47]. Considering the fact that a very small duration of interaction promoted well-being, constant interactions with a dog in one’s home may provide even greater benefits. Further, interactions between adolescents and dogs are not limited to therapy and could be promoted in school and other various situations where pets are allowed. On the other hand, when living together with cats, people should be aware of the risk of *T. gondii*. Based on previous studies, we believe that in order to prevent this disease, it would be helpful for the respective owners to keep his/her cat indoors, limit its hunting, clean litter pan daily, and dispose of feces in the toilet while wearing disposal gloves [48]. A recent study attempted to develop a vaccine for *T. gondii* [49]. These challenges may lead to a better life for cats and humans. One of the strengths in our study is overcoming the methodological limitations which were suggested in the systematic review [14]. Firstly, the sample size of TTC was large (*N* = 2584), while those in the previous studies were small (*N*: 15–1541). Secondly, samples in most of the previous studies were homogeneous and self-selected; however, the TTC sample was population-based. Thirdly, the main study design of previous studies was cross-sectional (13/22), while our TTC study was longitudinal with a prospective design, which enabled us to analyze temporal direction. Fourth, TTC had several demographic and socioeconomic variables to adjust covariates. We adjusted for multiple confounders which were applied in the previous studies. Fifth, dogs and cats were separately addressed in this study to investigate the varying effects of different pet types.

There are nevertheless some limitations. Firstly, TTC was not initially designed to study pet ownership, so we missed the data of pet ownership before and after age 10. Adolescents who lost their pets before age 10 or who owned pets after age 10 might be coded as non-owners in this study. We also did not have the information on the age of pets, duration of pet ownership, amount of time spent with the pet, which family member takes most care of the pet, and the strength of the attachment to the pet. Involvement with pets may be reflected in the strength of the ownership effect. These factors should be considered in future studies. Secondly, pets are not only limited to dogs and cats. Other pet species may also have a different effect. Thirdly, we adjusted for all confounders mentioned in the previous studies, however, the possibility of another confounding factor exists. Fourth, we did not examine whether the prediction of well-being from dog ownership and cat ownership is limited to adolescents in Tokyo, which is an affluent urban area in a developed Asian country. Geographical or cultural differences in ownership may exist. Fifth, we did not consider the subjective or general health of pet owners. Subjective health was controlled in the previous wellbeing study [30]. In the systematic review of pets and adolescents, the possibility exists that families with children experiencing difficulties in health or development may tend to have more or fewer pets [14]. Future studies should examine problems in health and/or developmental difficulties. Sixth, we did not differentiate whether the pet owner was the child, another family member, or the family as a whole in this study. Future studies can consider whether this difference could also have an effect.

To the best of our knowledge, this was the first large-sampled, longitudinal, population-based study which has investigated the different effects of dog and cat ownership on adolescent’s well-being while adjusting for covariates and analyzing the differences among these two species. When compared to the well-being of non-dog/cat owners, dog ownership predicted positive adolescents’ well-being, and cat ownership predicted negative adolescents’ well-being. The well-being of dog owners was maintained through the study period from 10–12 years, whereas the well-being of cat owners seemed to decline through the study period from 10–12 years.

## 5. Conclusions

Dog ownership and cat ownership differently predicted adolescents’ well-being. Dog ownership had a positive effect on adolescents’ well-being compared to no dogs, however, cat ownership had a negative effect compared to no cats. The well-being trajectory of dog owners was maintained through adolescence, while that of cat owners declined.

## Figures and Tables

**Table 1:** Demographic characteristics of participants (N = 2584).

		All		Dog owners		Cat owners		Non-dog/cat owners	
		Number/*mean*	%/*SD*	Number/*mean*	%/*SD*	Number/*mean*	%/*SD*	Number/*mean*	%/*SD*
Sex	Female	1218	47.1%	119	46.9%	49	45.0%	1053	47.2%
	Male	1366	52.9%	135	53.1%	60	55.0%	1177	52.8%
Age (months)		122	3.28	122	3.21	121	3.26	122	3.29
Well-being	at age 10	79.06	16.61	79.42	16.83	80.04	15.65	78.98	16.63
	at age 12	75.14	18.88	77.53	17.60	69.69	21.06	75.11	18.87
Parental age	Mother	41.97	4.15	41.48	4.27	42.40	4.11	42.00	4.14
	Father	44.12	5.17	43.40	5.12	45.26	5.00	44.16	5.19
Educational level of mother	High school or less	411	15.9%	44	17.3%	15	13.8%	352	15.8%
	2-year college	1150	44.5%	129	50.8%	53	48.6%	973	43.6%
	4-year university	932	36.1%	71	28.0%	38	34.9%	826	37.0%
	Graduate university	91	3.5%	10	3.94%	3	2.8%	79	3.5%
Educational level of father	High school or less	470	18.2%	59	23.2%	28	25.7%	385	17.3%
	2-year college	360	13.9%	33	13.0%	23	21.1%	305	13.7%
	4-year university	1444	55.9%	137	53.9%	44	40.4%	1268	56.9%
	Graduate university	310	12.0%	25	9.8%	14	12.8%	272	12.2%
Annual household income (10,000 yen)	0–299	64	2.5%	7	2.8%	4	3.7%	53	2.4%
	300–599	636	24.6%	65	25.6%	39	35.8%	535	24.0%
	600–999	1095	42.4%	96	37.8%	38	34.9%	964	43.2%
	1000+	789	30.5%	86	33.9%	28	25.7%	678	30.4%
Number of siblings		1.16	0.79	1.12	0.78	1.07	0.84	1.16	0.78

**Table 2:** Multiple linear regression analysis for well-being at age 12.

	Unadjusted					Adjusted ^1^				
	*B*	95%CI			*p*	*B*	95%CI			*p*
Dog ownership	2.61	0.17	-	5.05	0.036	2.45	0.19	-	4.71	0.033
Cat ownership	−5.65	−9.26	-	−2.03	0.002	−6.14	−9.49	-	−2.79	0.000

^1^ Adjusted for well-being at age 10, sex, age (months), parental age, parental educational level, annual household income, and the number of siblings.
